# Experimentally Deduced Criteria for Detection of Clinically Relevant Fusion 3′ Oncogenes from FFPE Bulk RNA Sequencing Data

**DOI:** 10.3390/biomedicines10081866

**Published:** 2022-08-02

**Authors:** Elizaveta Rabushko, Maxim Sorokin, Maria Suntsova, Alexander P. Seryakov, Denis V. Kuzmin, Elena Poddubskaya, Anton A. Buzdin

**Affiliations:** 1 Laboratory for Clinical and Genomic Bioinformatics, Institute of Personalized Oncology, I.M. Sechenov First Moscow State Medical University, 119991 Moscow, Russia; elnrabush@gmail.com (E.R.); sorokin@oncobox.com (M.S.); suntsova@oncobox.com (M.S.); podd-elena@ya.ru (E.P.); 2 Department of Biological and Medical Physics, Moscow Institute of Physics and Technology, 141701 Dolgoprudny, Russia; kuzmin.dv@mipt.ru; 3 OmicsWay Corp., 340 S Lemon Ave, 6040, Walnut, CA 91789, USA; 4 Medical Holding SM-Clinic, 125130 Moscow, Russia; alseryakov@yandex.ru; 5 Shemyakin-Ovchinnikov Institute of Bioorganic Chemistry, 117997 Moscow, Russia; 6 PathoBiology Group, European Organization for Research and Treatment of Cancer (EORTC), 1200 Brussels, Belgium

**Keywords:** fusion oncogene, receptor tyrosine kinase, RNA sequencing, RNAseq, FFPE, tumor molecular diagnostics, clinical oncology

## Abstract

Drugs targeting receptor tyrosine kinase (RTK) oncogenic fusion proteins demonstrate impressive anti-cancer activities. The fusion presence in the cancer is the respective drug prescription biomarker, but their identification is challenging as both the breakpoint and the exact fusion partners are unknown. RNAseq offers the advantage of finding both fusion parts by screening sequencing reads. Paraffin (FFPE) tissue blocks are the most common way of storing cancer biomaterials in biobanks. However, finding RTK fusions in FFPE samples is challenging as RNA fragments are short and their artifact ligation may appear in sequencing libraries. Here, we annotated RNAseq reads of 764 experimental FFPE solid cancer samples, 96 leukemia samples, and 2 cell lines, and identified 36 putative clinically relevant RTK fusions with junctions corresponding to exon borders of the fusion partners. Where possible, putative fusions were validated by RT-PCR (confirmed for 10/25 fusions tested). For the confirmed 3′RTK fusions, we observed the following distinguishing features. Both moieties were in-frame, and the tyrosine kinase domain was preserved. RTK exon coverage by RNAseq reads upstream of the junction site were lower than downstream. Finally, most of the true fusions were present by more than one RNAseq read. This provides the basis for automatic annotation of 3′RTK fusions using FFPE RNAseq profiles.

## 1. Introduction

Chromosomal rearrangements resulting in the formation of transcribed fusion genes are frequent driver mutations in different cancer types [1]. Receptor tyrosine kinase (RTK) gene fusions are of particular interest to study since they can be a source of druggable chimeric proteins with increased tyrosine kinase activity [2]. For example, imatinib is a tyrosine kinase inhibitor active on a BCR-ABL1 fusion and is FDA approved to treat chronic myeloid leukemia patients with the corresponding translocation. Moreover, entrectinib and larotrectinib obtained FDA approval as the tissue-agnostic drugs for the treatment of tumors positive for *NTRK1-3* gene fusions, regardless of the cancer type [3,4]. Pan-cancer drug target RTK fusions also include the moieties of *ALK*, *ERBB2*, *FGFR1-4*, *MET*, *RET*, and *ROS1* genes [5]. Hence, the detection of specific gene fusions in individual cancer samples is a crucial step in effective treatment selection, prognosis, and tumor molecular classification. 

Formalin-fixed paraffin-embedded (FFPE) tissue blocks are the most common way of preserving cancer tissue biomaterials in clinical biobanks because of their relative simplicity, robustness, and cost-effectiveness [6]. However, identification of fusion oncogenes from FFPE materials is a non-trivial task because there are several alternative methods available that can produce results which may contradict each other. The method of fluorescent in situ hybridization (FISH) utilizes hybridization of specific labeled DNA probes and allows the detection of chromosomal rearrangements with nearly a 1 megabase resolution in both fresh and fixed tumor tissue samples [7]. For example, FISH is a standard method for detecting the *BCR-ABL1* translocation (Philadelphia chromosome) [8]. Nevertheless, FISH diagnostics can neither offer information about functional activities of fusion genes and preservation of an open reading frame, nor can they allow detection of the non-canonical or unknown fusions. In addition, FISH is poorly sensitive to the detection of local intrachromosomal rearrangements such as for the case of *FGFR2* fusion genes [9].

The immunohistochemistry (IHC) method is simple and cost-effective, and its use has been approved by the US FDA for the detection in lung cancer of *ALK* [10] and *ROS1* rearrangements [11]. However, IHC senses not the chimeric gene product itself, but rather the expression level of the respective tyrosine kinase domain, and a stronger IHC signal can be also due to a stronger expression of the intact, non-fusion RTK gene. 

Furthermore, reverse transcription PCR (RT-PCR) is a standard method for detecting the presence of chimeric transcripts including fusion oncogenes, and several kits are commercially available to detect *NTRK1-3*, *ALK*, *RET*, and *ROS1* fusion RNAs in FFPE biosamples [8,12]. Another method—digital multiplex PCR—measures the presence of fusion RNA from as little as 1pg of RNA in miniature droplets, each encompassing a single RNA molecule, allowing a “yes–no” assessment of PCR results [13]. However, all those PCR methods are sensitive only when both fusion partners are known and included within specific panels, and not informative otherwise, e.g., for the RTK fusions with rare or new partners. 

Further, a number of targeted DNA sequencing approaches have been developed to detect structural rearrangements of chromosomes that lead to the formation of fusion RTK genes. For example, Agilent has developed a panel for the detection of both known and not yet described chimeric RTK genes, consisting of biotin-labeled DNA probes that are complementary to the intron and exon sequences of the genome, which are known breakpoints in the formation of chimeric genes [14]. However, most fusions occur in intron sequences, which often contain repetitive sequences, and are sequenced less efficiently than coding sequences—especially for the fragmented DNAs extracted from FFPE samples. This can lead to false negative results, e.g., when the presence of the chimeric gene is confirmed by another method such as FISH or RNA sequencing. Davies et al. compared three approaches to detect *ROS1* gene rearrangements in lung cancer [15]. Targeted DNA sequencing did not detect 4 out of 18 chimeric genes confirmed by alternative approaches. Similarly, Benayed et al. found that 14% of tumors with confirmed RTK chimeric genes were not detected when analyzed by the US FDA-approved MSK-IMPACT panel [16]. 

Finally, RNA sequencing (RNAseq) is a method that has several advantages over the previous approaches. Except for the RTK fusions, in one experimental procedure it is also possible to analyze the expression level of genes linked with the effectiveness of cancer drugs [17,18], activation or inhibition of various molecular pathways [19,20], and for the assessment of tumor mutation burden [21], and still for the FFPE biosamples. The detection of fusion RTK transcripts from RNAseq reads has a number of advantages, e.g., only transcriptionally active entries are identified, thereby filtering out the passenger mutations which are not rare in cancers for RTK fusion genes as well [22]. Second, with RNAseq both parts of a fusion gene can be identified at once—equally effective for both known and previously unknown fusion partners. In addition, integrity of an open reading frame can be easily assessed in the fusion gene product, as well as the presence of an intact kinase domain. Compared to the targeted RNAseq which interrogates RNAs captured by oligonucleotide hybridization or PCR primer panels specific for RTKs of interest, bulk RNAseq has the advantage of detection of both targeted and not specifically targeted types of fusion transcripts [23,24,25,26]. This advantage is especially important considering emerging pairs of complementary diagnostic fusion oncogenes and drugs on the way to acceptance and clinical use [27].

Multiple bioinformatic algorithms have been developed to identify candidate fusion transcripts from RNAseq data, aiming to find discordantly mapping chimeric reads [28]. Although there is still no gold standard algorithm for fusion detection, recently published comparisons of different tools have revealed STAR-Fusion and Arriba as being some of the most effective prediction methods [28,29]. However, RNA-seq data analysis for fusion detection leads to a great number of false-positives due to artifacts arising during library preparation because of the reverse transcriptase template switching activity, cross-ligation of RNAs, and difficulties in short-reads sequence alignment [30]. Thus, the manual curation and experimental confirmation of predicted fusions is required, enlarging the time and sample amount necessary for biomarker analysis. Another way to decrease the number of false-positive results is implementation of stringent filters, often leading to the discarding of driver fusions and loss of disputable events in RNA-seq data. The loss of important chimeric transcripts is particularly significant when analyzing FFPE samples, where more degraded RNA leads to an increased number of controversial reads, which are apparently excluded after filtration. 

Thus, the development of new criteria enabling researchers to algorithmically distinguish true fusion from artefacts is an essential step in OMICS-based precision oncology improvement. In this study, we attempted to formulate a new approach for algorithmic fusion verification by defining true fusions’ specific parameters using FFPE tumor bulk RNAseq profiles. We investigated clinically relevant RTK fusion genes, namely *ABL1*, *ALK*, *ERBB2*, *FGFR1-4*, *NTRK1-3*, *RET*, and *ROS1*, because they are among the most frequent druggable cancer molecular biomarkers. To this end, we applied STAR-Fusion software to analyze experimental RNAseq data obtained for 764 cancer patient FFPE samples in the Oncobox NCT03724097 clinical trial, 96 leukemia samples, and 2 cell line samples. We then manually curated and experimentally validated by RT-PCR and Sanger sequencing RTK fusion genes predicted from preliminary STAR-Fusion output files. Then we compared structural and gene expression features of experimentally confirmed and non-confirmed fusions. For the confirmed 3′ RTK fusions, we observed the following features distinguishing “true” active cancer fusions from the artifacts. Both moieties were in-frame, and the tyrosine kinase domain was preserved. RTK exon coverage by RNAseq reads upstream of the junction site was lower than downstream. Finally, most of the true fusions were present by more than one RNAseq reads. Taken together, this provides the basis for automatic annotation of 3′ RTK fusions using FFPE RNAseq profiles. Thus, we suggest a new approach for facile verification of bulk RNAseq predicted fusions without the need for experimental validation and stringent filtering criteria application, especially adapted for both fresh and archival FFPE samples.

## 2. Materials and Methods

### 2.1. Biosamples and RNA Sequencing

Tumor tissue biosamples were obtained from 764 solid cancer patients included in the clinical trial Oncobox (Clinicaltrials.gov ID NCT03724097), 96 leukemia patients, and 2 cell lines. The biosamples were FFPE tumor tissue blocks (one block per patient) containing at least 50% cancer cells for solid tumors and bone marrow biopsies for leukemias that were evaluated by a pathologist to confirm the diagnosis. In addition, we also explored a set of control cell lines. Leukemia samples and cell lines were stabilized in RNAlater solution (Qiagen, Hilden, Germany) and then stored at −70 °C.

After obtaining a sufficient amount of biomaterial for RNA sequencing, the biosamples were returned to the clinical laboratories involved. The patients were 348 men and 496 women (16 with unknown status), the mean age was 51.1 years old (range 1.5–84 years old), and the samples were clinically annotated with gender, age, and diagnosis (Supplementary Table S1).

For every patient biosample, written informed consent to participate in this study was obtained from the patient or his/her legal representative. The consent procedure and the design of the study were guided and approved by the local ethical committees of the Vitamed Clinic (Moscow) and I.M. Sechenov First Moscow State Medical University.

RNAs were extracted, quantified, and sequencing libraries were created according to [18]. RNA sequencing was performed using an Illumina NextSeq 550 engine for single-end sequencing, with at least 30 million raw reads per sample, for a 50 bp read length. A data quality check was performed on an Illumina SAV, and de-multiplexing was performed with Illumina Bcl2fastq2 v 2.17 software. All 860 primary tumor RNA profiles had at least 2.5 million uniquely HGNC gene-mapped reads as recommended for the current protocol of RNAseq reads processing [31].

Following fusion transcripts calling, the further availability of biomaterials was revised for the patient samples where putative RTK fusion transcripts were detected. Where available, the samples were obtained again for the patients who signed new written informed consent to participate in the study including confirmation of RTK fusion transcripts by reverse transcription PCR and Sanger sequencing of the obtained PCR products. This consent procedure and the design of the study were approved by the I.M. Sechenov First Moscow State Medical University local ethical committee. The experimental RTK fusion validation dataset included 5 lung cancer samples, 4 acute leukemia samples, 2 ovarian cancer samples, 2 breast cancer samples, and 1 sample for each of the breast fibrosarcoma, Kaposi sarcoma, pseudomyxoma peritonei, non-gestational choriocarcinoma, glioblastoma, epithelioid sarcoma, pancreatic cancer, and cell lines BT474 and A549. Overall, a new set of biosamples was obtained from 8 male and 12 female patients. The mean age was 44 years old (range 2–65 years old) (Supplementary Table S2). 

### 2.2. Bioinformatic Identification of RTK Fusion Transcripts 

Fusion transcripts were initially screened using STAR-Fusion software [28] in RNAseq profiles for the 860 above cancer samples and two cancer cell lines according to [26]. Preliminary files containing fusion candidates were generated, and the corresponding RNA sequencing reads with *ABL1, ALK, ERBB2, FGFR1-4, NTRK1-3, RET*, and *ROS1* genes were extracted. The output data were manually inspected using UCSC BLAT and the UCSC Browser (https://genome.ucsc.edu/ accessed on 1 January 2022) to interrogate fusion candidates according to the following criteria: (i) does the read cover an exon junction of two different known mapped transcripts, (ii) if the junction point exactly matches the exon termini of known genes with established splice sites, (iii) if both transcripts are in the same orientation within a putative fusion RNA.

### 2.3. Experimental Validation of RTK Fusion Transcripts 

Total RNA was isolated from formalin-fixed paraffin-embedded (FFPE) tissue sections using the RNeasy FFPE Kit (Qiagen, Valencia, CA, USA). RNA isolation from samples stored in RNAlater was carried out using the QIAGEN Rneasy Kit (Qiagen, Valencia, CA, USA), following the manufacturer’s protocol. To confirm the presence of the fusion transcripts, we performed reverse transcription PCR (RT-PCR) with oligonucleotide primers designed to specifically anneal to either side of the putative RTK fusion transcript breakpoint (Supplementary Table S3). First strand cDNA was synthesized from 0.5 to 1 µg total RNA using the MMLV RT kit (Evrogen, Moscow, Russia); cDNA was then amplified using the qPCRmix-HS SYBR kit (Evrogen, Moscow, Russia), and real-time RT-PCR was carried out using CFX96 Touch™ real-time PCR equipment (BIO-RAD, Hercules, CA, USA) in a 25 μL final volume containing 1× qPCRmix-HS SYBR, 25 pmol of primer pair (Table 1), and 1 μL of cDNA per reaction. PCR products were then assessed using melting curve analysis and by electrophoresis in 2% agarose gels. For the PCR products obtained, Sanger sequencing was carried out by Evrogen (Moscow, Russia) in both directions using the same primers as for RT-PCR amplification.

### 2.4. Exon Coverage Calculation

To assess exon coverage of potential fusion partner genes, we used BEDtools multicov v2.26.0 software with default parameters. The input files were BAM (Binary Alignment/Map) files resulting from RNA sequencing and BED (Browser Extensible Data) files with the exonic coordinates of the transcripts included in the MANE (Matched Annotation between NCBI and EBI) project. The read counts obtained were double normalized on (i) exon length and (ii) sequencing depth of a particular experimental sample. The code used for the analysis and the accompanying data are available at https://gitlab.com/erabushko/fusionscriteria/ accessed on 12 July 2022.

## 3. Results

The functional roles of 5′ and 3′ fusion partners of RTK genes may be totally different. The 5′ partners (RTK on 3′ end) are frequently transcriptional drivers with strong promoters [33], while the 3′ partners (RTK on 5′ end) usually cause ligand-independent dimerization of RTK domains [34]. In our recent study, we identified a novel cancer in-frame fusion transcript with the kinase domain on the 3′ end, with NCOA4-RET in an FFPE papillary thyroid cancer sample [25]. In this sample, the detailed investigation revealed significant disproportion in RET gene exon RNAseq reads coverage: it was statistically significantly higher downstream to the fusion site (*t*-test, *p*-value = 0.0011). Thus, in this study, we aimed to specifically investigate whether the apparent disbalance of the exon coverage in the 3′ fusion partner gene between its upstream and downstream parts relative to the fusion site is a general phenomenon that could be employed to discriminate true 3′ RTK fusions using FFPE RNAseq data. 

We took into the analysis cancer fusions of RTK genes ABL1, ALK, ERBB2, RET, ROS1, NTRK1-3, and FGFR1-4 because they are important molecular biomarkers guiding prescription of specific targeted therapeutics. We first aimed to bioinformatically identify RTK fusions in the clinical sampling of 764 FFPE RNAseq profiles obtained from the patients included in the clinical trial Oncobox in 2018–2021, 96 leukemia samples, and 2 cell lines. For all solid tumor samples, the RNAseq reads were obtained from FFPE materials and were relatively short (~50 nt long). Due to high fragmentation of RNA and consequently short reads, FFPE samples are excluded from the protocols recommended by most software developers including STAR-Fusion, the most effective fusion transcript calling tool according to a comparative study [28]. However, in this study, we attempted to adopt the STAR-Fusion calling pipeline and performed a manual check of intermediate fusion calling results, yet the final software output was the probable lack of fusions in all the samples. However, we reviewed intermediate STAR-Fusion files to identify putative fusion candidates and found 989 such candidates in 351 samples. We then manually inspected all such candidates with UCSC BLAT and UCSC Browser tools using the following criteria for the putative RTK fusion: (i) RNAseq read has to cover the exon junction of two different known transcripts, (ii) both fusion partner moieties must be unambiguously mapped on known human transcripts, (iii) the junction point has to exactly match the exon termini of genes with established splice sites, and (iv) both fusion moieties must be in the same transcriptional orientation. Out of the total 989 candidates, we identified only 36 putative RTK fusion transcripts (3.6% successful candidates, Supplementary Table S4). Among them, for eight putative fusions, we identified more than one non-duplicated chimeric reads (Supplementary Table S4).

**Figure 1:**
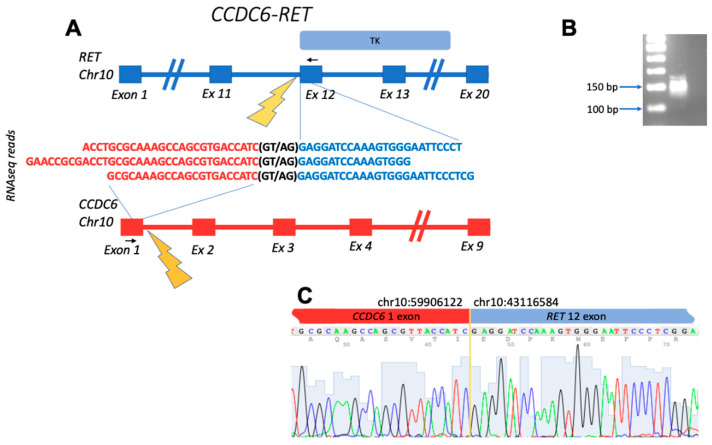
Schematic representation of the *CCDC6-RET* fusion transcript identified: (**A**) gene structures upstream and downstream of fusion site; (**B**) electropherogram of RT-PCR product obtained with primers complementary to the fusion moieties. The deduced PCR product size is 143 bp long; (**C**) Sanger sequencing of RT-PCR product confirms the fusion of exon 1 of *CCDC6* with exon 12 of *RET*. Black arrows denote position of PCR primers. TK, tyrosine kinase domain within the structure of *RET*.

However, not all putative RTK fusions may be “true” because the chimeric reads may be the result of reverse transcriptase template switching and other events in the library preparation leading to related artifacts [35]. Thus, the experimental validation is a crucial step in the fusion discovery. The biomaterials for experimental fusion confirmation were available for 20 samples (matching for 25 individual fusions), and we used them in a series of reverse transcription polymerase chain reaction (RT-PCR) validation experiments with primer pairs complementary to either side of the fusion breakpoints, and subsequent Sanger sequencing was carried out for the final verification. The pipeline of experimental fusion validation is schematized in Figure 1. Totally, in such a way we experimentally confirmed 10 RTK fusions in five cancer samples (Table 1). Thus, only 10/25 bioinformatically deduced fusions that could be validated were true chimeric transcripts, whereas the other 60% most likely represented RNAseq library preparation artifacts.

**Figure 2:**
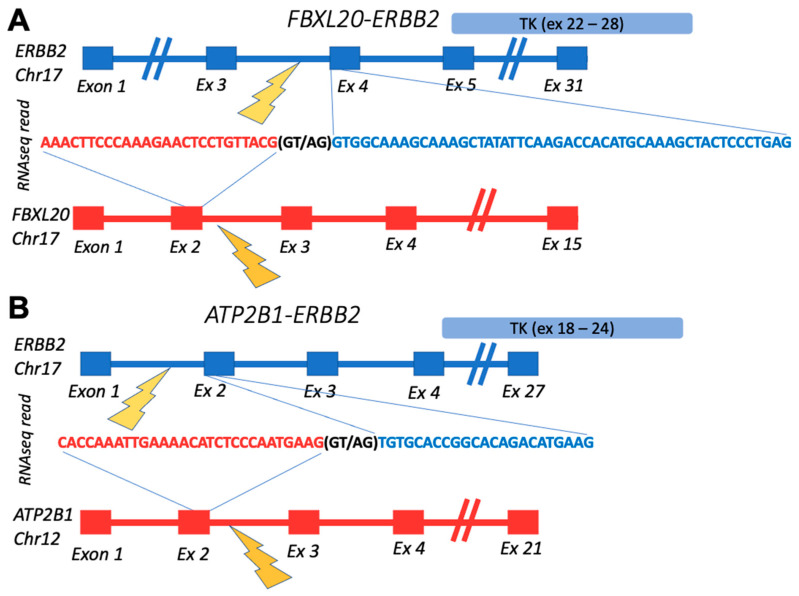
Schematic representation of fusion transcripts identified in BC105 sample: (**A**) gene structures upstream and downstream of *FBXL20-ERBB2* fusion site; (**B**) gene structures upstream and downstream of *ATP2B1-ERBB2* fusion site. TK, *ERBB2*-encoded tyrosine kinase domain.

The confirmed fusions were enriched by the “in-frame” status, which is crucial for retaining functions of their tyrosine kinase domains (Table 1). The out-of-frame confirmed transcript represented *FBXL20-ERBB2* fusion from the breast cancer sample BC105 which also had in-frame *ATP2B1-ERBB2* fusion with the same RTK partner (Figure 2). Interestingly, these fusions reads’ 3′-moieties, matching *ERBB2* gene parts, aligned to different *ERBB2* transcripts. While the *ERBB2* part of in-frame *ATP2B1-ERBB2* fusion mapped to the ENST00000269571.10 (https://www.ensembl.org/ accessed on 30 December 2021) transcript selected in MANE (the Matched Annotation from the NCBI and EMBL-EBI) project, which is chosen as the source of exon coordinates in this study, the *ERBB2* part of the out-frame *FBXL20-ERBB2* chimeric read matched to 5′-untranslated region of the ENST00000584601.5 transcript (https://www.ensembl.org/ accessed on 30 December 2021) (Figure 2).

**Figure 3:**
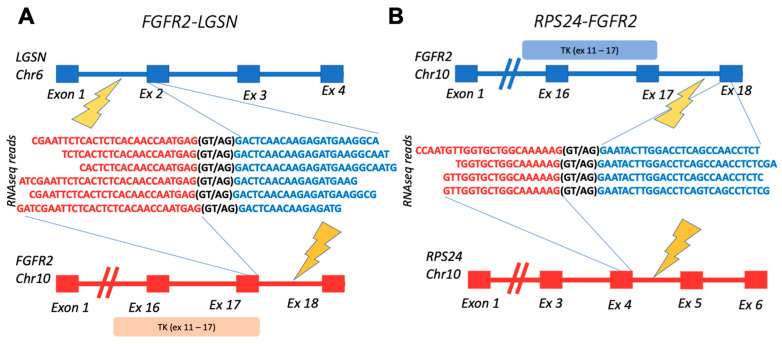
Schematic representation of fusion transcripts identified in OC11 sample: (**A**) gene structures upstream and downstream of *FGFR2-LGSN* fusion site; (**B**) gene structures upstream and downstream of *RPS24-FGFR2* fusion site. TK, *FGFR2*-encoded tyrosine kinase domain.

Interestingly, all but one confirmed fusion had an RTK partner sequence on the 3′ end. The exceptional case was the *FGFR2-LGSN* fusion from the ovarian cancer sample OC11. In the same sample, we also detected another fusion with FGFR2 at the 3′ terminus: *RPS24-FGFR2*, thus suggesting that *FGFR2* could be included in the DNA rearrangement hotspot in that cancer sample (Figure 3).

Except for the single above case of *RPS24-FGFR2* fusion, the other confirmed fusions most likely retained their tyrosine kinase domains (Table 1).

In addition, 7 out of 10 experimentally validated fusions had more than one chimeric RNAseq read, except for the well-known *BCR-ABL1* fusion detected in one of the leukemia samples and two variants of *SLC34A2-ROS1* detected in one of the lung cancer samples, which had only one chimeric read. Meanwhile, all the non-confirmed fusions were supported by only one chimeric read. Among the experimentally unexplored putative fusions, there was only one represented by more than one (eight) reads, i.e., for the *CNTNAP3-NTRK2* transcript in the glioblastoma N63 sample. 

Most of the non-confirmed fusions were also out-of-frame and lacked the tyrosine kinase domain with the exception of two cases: *KIF27-NTRK2* in the lung cancer sample LuC11 and *DOCK1-FGFR2* in the pancreatic cancer sample PC24 (Table 1).

For confirmed, non-confirmed, and experimentally unexplored putative fusions, we also analyzed presence of the chimeric transcripts with the same fusion partners in the most comprehensive related public databases ChimerDB (http://www.kobic.re.kr/chimerdb/ accessed on 21 June 2022), ChiTaRS (http://chitars.md.biu.ac.il/ accessed on 21 June 2022), and TumorFusions (https://tumorfusions.org/ accessed on 21 June 2022) (Table 1). Among the confirmed fusions, 7/10 were represented in at least one of these databases, versus 0/15 for the non-confirmed fusions, and 1/11 for the experimentally unexplored fusions (Table 1). 

**Figure 4:**
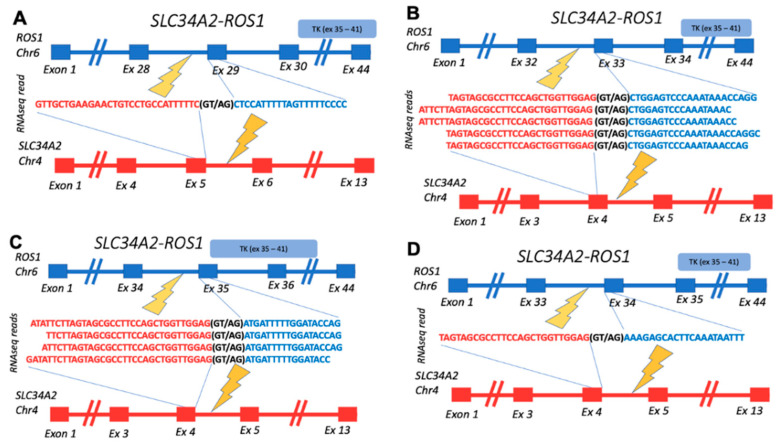
Schematic representation of *SLC34A2-ROS1* fusion transcripts identified: (**A**) gene structures upstream and downstream of fusion site for transcript *variant 1* (in-frame); (**B**) gene structures upstream and downstream of fusion site for transcript *variant 2* (in-frame); (**C**) gene structures upstream and downstream of fusion site for transcript *variant 3* (in-frame); (**D**) gene structures upstream and downstream of fusion site for transcript *variant 4* (out-frame). TK, *ROS1*-encoded tyrosine kinase domain.

Among the confirmed fusions, several entries were also previously published in the scientific reports. Among them are *BCR-ABL1*, known as Philadelphia chromosome, which is the first characterized cancer chimeric transcript which is frequently found in leukemias [36]; detected here in acute lymphoblastic leukemia. *CCDC6-RET* is the most common *RET* fusion in papillary thyroid cancer, which was also identified in lung cancer, acute lymphoblastic leukemia, and other cancers [37]; detected here in breast fibrosarcoma. *SLC34A2-ROS1* was previously detected in different cancer types, with the greatest prevalence in lung adenocarcinoma, dedifferentiated liposarcoma, and breast invasive ductal carcinoma [38]; detected here in lung cancer. In this study, we detected four variants of this fusion gene, all with the retained tyrosine kinase domain and 3/4 in-frame (variants 1–3) (Figure 4).

The *FGFR2-LGSN* transcript has been previously detected in cholangiocarcinoma, however it is not yet functionally characterized [32]; detected here in ovarian cancer. Interestingly, the latter chimeric transcript was absent from the related fusion databases (Table 1).

Finding criteria specifically for the verified fusions that may help algorithmically distinguish between the true and artifact RTK fusions predicted after FFPE RNAseq reads is a crucial task for skipping the need for RT-PCR experimental validation and, therefore, for broad-scaling and translating RNAseq analyses into the clinic. Thus, we then analyzed exon coverage patterns of 3′ and 5′ fusion partner genes for both confirmed and non-confirmed fusions. We did not find any specific features of the 5′ partner gene exon coverage.

**Figure 5:**
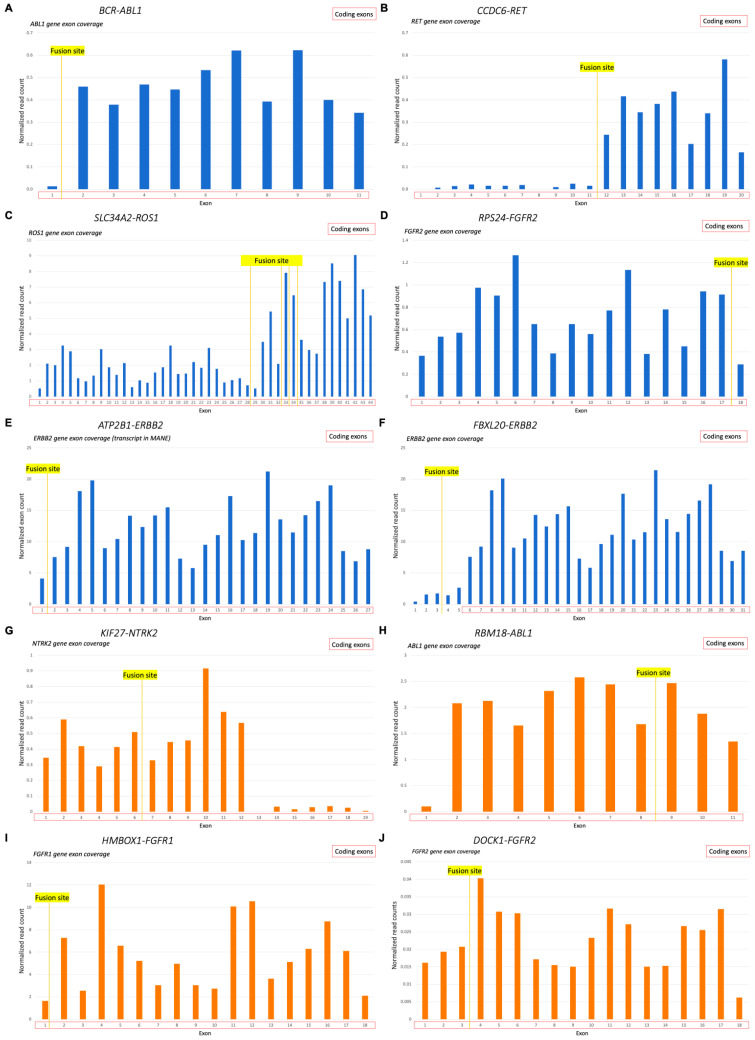
The 3′ fusion partner gene exon coverage, investigated for the 3′RTK fusion transcripts: (**A**) length-normalized RNAseq reads coverage of *ABL1* gene in sample AL98 (*BCR-ABL1* fusion); (**B**) length-normalized RNAseq reads coverage of *RET* gene in sample FS1 (*CCDC6-RET* fusion); (**C**) length-normalized RNAseq reads coverage of *ROS1* gene in sample LuC46 (SLC34A2-ROS1 fusion, all variants); (**D**) length-normalized RNAseq reads coverage of *FGFR2* gene in sample OC11 (*RPS24-FGFR2* fusion); (**E**) length-normalized RNAseq reads coverage of *ERBB2* gene in sample BC105 (*ATP2B1-ERBB2* fusion); (**F**) length-normalized RNAseq reads coverage of *ERBB2* gene in sample BC105 (*FBXL20-ERBB2* fusion); (**G**) length-normalized RNAseq reads coverage of *NTRK2* gene in sample LuC11 (*KIF27-NTRK2* fusion); (**H**) length-normalized RNAseq reads coverage of *ABL1* gene in sample J11 (*RBM18-ABL1* fusion); (**I**) length-normalized RNAseq reads coverage of *FGFR1* gene in sample EpS1 (*HMBOX1-FGFR1* fusion); (**J**) length-normalized RNAseq reads coverage of *FGFR2* gene in sample PC24 (*DOCK1-FGFR2* fusion). (**A**–**F**), verified fusions (blue); (**G**–**J**), non-verified fusions (orange).

However, the exon coverage patterns of 3′ partner genes were clearly distinct for the samples with verified RTK 3′ fusions. We observed a significant difference in the exon coverage when comparing downstream and upstream parts of the 3′ fusion partner gene relative to the fusion site: exons in the downstream part had statistically significantly higher length-normalized levels of RNAseq reads (*t*-test, *p* < 0.05) (Figure 5). For the non-verified fusions, no statistically significant difference was detected in exon coverage of genes’ 3′ fusion partners, even for the in-frame fusions with an intact kinase domain (Figure 5).

**Figure 6:**
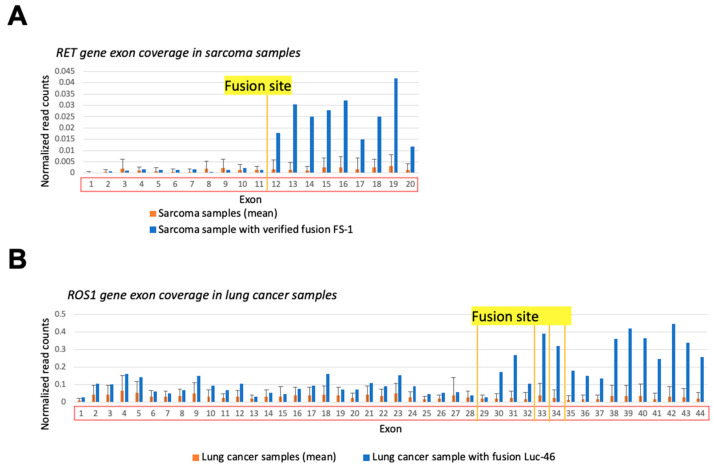
Gene exon coverage comparison in sample with verified 3′RTK fusion and the other cancer samples of the same type. Double normalized exon coverage by RNA sequencing reads is shown: (i) by exon length and (ii) by overall gene expression level in a sample. (**A**) RET gene exon coverage in FS-1 sample (blue) and in all sarcoma samples (orange). (**B**) ROS1 gene exon coverage in LuC46 sample (blue) and in all lung cancer samples (orange).

In principle, uneven exon coverage may be associated with technical or biological causes not necessarily related to the fusion formation. For instance, the sequence-specific RNA degradation pattern or prevalence of a specific transcript isoform can theoretically affect exon coverage. To exclude such factors, we compared the exon coverage in a sample where fusion was detected with the average exon coverage in the other samples of the same cancer type that had no such fusion transcript reads (Figure 6). Comparisons of the exon coverage levels upstream/downstream to the fusion site in the control samples without detected fusion transcripts did not result in statistically significant differences (Figure 6A,B, orange), thus proving the connection between exon coverage pattern and presence of a fusion (Figure 6A,B, blue). Specifically, the coverage of the exons downstream to the fusion site in the sample with fusion was higher than the 95th percentile of the same exons’ mean coverage in the control group, while the coverage of upstream exons was fitting within the 95% confidence interval (CI) of the exon coverage level in samples without fusions.

However, when analyzing *ERBB2* gene exon coverage in the BC105 sample having verified fusion *ATP2B1-ERBB2*, we found no fusion-specific expression patterns. The coverage of all *ERBB2* exons in the tumor sample with fusion was within the 95% CI of the exon coverage in breast cancer samples, as well as above the 95th percentile exon coverage of normal breast tissue samples (data not shown). Formation of a fusion detected here may reflect massive amplification of *ERBB2* in HER2-positive breast tumors, thus explaining the increased expression of all exons, even those not included in the fusion which represent only one truncated copy of *ERBB2* in comparison to many amplified full-size gene copies.

**Figure 7:**
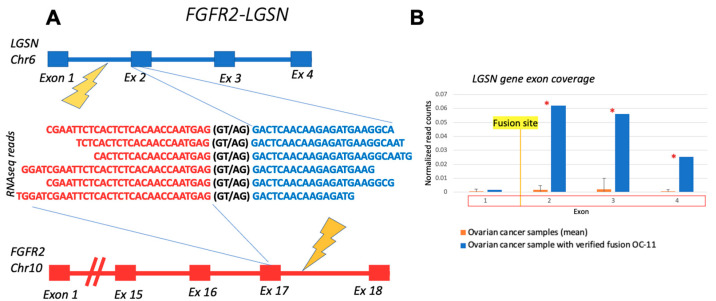
Schematic representation and RNAseq reads coverage of *FGFR2-LGSN* fusion transcript identified in OC11 sample: (**A**) fusion partner gene structures upstream and downstream of fusion site; (**B**) *LGSN* exon coverage by normalized RNA sequencing reads in OC-11 sample (blue) and in control ovarian cancer samples without fusion (orange). Double normalized exon coverage by RNA sequencing reads is shown: (i) by exon length and (ii) by overall gene expression level in a sample. *—the coverage of exon in OC-11 sample is higher than the 95th percentile of exon coverage in unrelated control ovarian cancer samples without fusion.

The 5′ RTK fusion *FGFR2-LGSN* that was verified here in the ovarian cancer sample OC11 has been previously detected in cholangiocarcinoma; however, it has not yet been functionally characterized. *LGSN*, a 3′-partner gene, is not generally expressed in ovarian cells. Therefore, the comparison of *LGSN* coverage in OC11 with its coverage in the other ovarian cancer samples demonstrated differential patterns (Figure 7). In the OC11 sample, the coverage of *LGSN* exons involved in the fusion transcript was significantly higher, i.e., higher than the 95th percentile of exon coverage in unrelated control ovarian cancer samples without fusion.

Furthermore, we suggested that the level of 5′-partner gene expression must be in accordance with the expression level of 3′-partner gene exons which were included in a fusion transcript. We examined whether the average coverage of 3′-partner exons, comprising fusion parts, better correspond to 5′ gene coverage by RNAseq reads in the experimentally confirmed vs. non-confirmed RTK fusions. However, among confirmed fusions, the concordance in both genes’ expression level was detected only for *CCDC6-RET* in the fibrosarcoma FS1 sample. Interestingly, only in the BC105 sample with *ERBB2* verified fusions the expression of 3′-partner gene *ERBB2* was significantly higher, while for the other eight confirmed fusions, 5′-partner gene greater coverage was observed. The increased *ERBB2* expression may be associated with massive gene amplification. Meanwhile, no consistent patterns of 5′/3′ expression levels’ correspondence were identified in samples with experimentally non-confirmed fusions. Thus, the comparison of 5′/3′-partner expression levels is a rather controversial criterion.

To conclude, we studied different fusion characteristics, namely, (i) the retention of ORF and (ii) the TK domain, (iii) presence in fusion DBs, (iv) statistically significant disbalance in 3′-gene exon coverage (greater RTK downstream reads), (v) number of unique chimeric reads (one/more than one), and (vi) the concordance in the expression of 5′/3′-moieties, to reveal their capacity of differentiating true and artifact experimentally verified fusions (Table 1). While the last parameter returned a rather questionable result for fusion verification, the others showed either high sensitivity (Sn), or specificity (Sp), or both (Table 1). The best combination of Sn and Sp was detected for the preservation of the tyrosine kinase domain (0.9 and 0.87, respectively), presence in fusion DBs (0.7 and 1), greater number of RTK reads downstream of the fusion site (0.78 and 1), and for the representation by more than one non-duplicated chimeric RNAseq reads (0.7 and 1). 

Only 30% of the confirmed fusions were positive for all five criteria. Meanwhile, none of the non-confirmed ones were positive for at least three parameters at once, and only two simultaneously could meet the two criteria. Thus, defining the threshold as at least two of five positive parameters present overall Sn 1 and Sp 0.87. Moreover, the threshold of at least three of five matched parameters present results of Sn 0,9 and Sp 1.

It should be noted that from the clinical point of view, only the fusions with an intact ORF and TK domain may seem relevant, since these are the signs of potentially active RTK chimeric proteins that could be inhibited by the respective RTK-specific drugs. For this group, the parameters (iii–v) slightly improved Sn: 0.71, 0.83, and 0.71, respectively; and retained Sp 1. Moreover, applying at least two of these three positive criteria lead to an overall Sn improvement to 0.86. 

Thus, we demonstrate here that combining different parameters improves overall quality of clinically relevant fusion identification.

## 4. Discussion

We analyzed the structure and expression features of bioinformatically predicted fusions with clinically relevant tyrosine kinase genes based on the experimental FFPE RNAseq expression data for 763 cancer samples, as well as for 96 leukemia samples and 2 cell lines. The “true” or “artifact” state of the fusions was validated in a series of RT-PCR experiments where the starting patient biomaterials were available for an additional testing. Most of the fusions had RTK moiety on the 3′ end. 

One of the experimentally verified 3′RTK fusions (*ATP2B1-ERBB2*) from breast cancer sample BC-105 has not been previously included in any of the three most comprehensive fusion databases (ChimerDB, ChiTaRS, and TumorFusions). However, both genes are known to form fusions with other partners. For instance, eight unique fusions with the *ATP2B1* gene are listed in the ChimerDB database, which were found in TCGA sequencing data but are not functionally characterized. The *ATP2B1* (ATPase plasma membrane Ca^2+^ transporting 1) gene encodes one of the plasma membrane type Ca^2+^ pumps or the Ca^2+^ ATPases (PMCAs) isoform. PMCA is responsible for the expulsion of Ca^2+^ from the cytosol into the extracellular space, to maintain a low intracellular Ca^2+^ concentration. Several studies investigated the *ATP2B1* role in breast cancer, and Varge et al. showed no significant alterations in *ATP2B1* expression in invasive breast cancer tissue samples [39]. However, more recently an association between tumor stage and *ATP2B1* was identified [40]. Meanwhile, *ERBB2* (erb-b2 receptor tyrosine kinase 2) is a member of the epidermal growth factor (EGF) receptor family genes encoding for well-known HER2 protein, which is known to play an essential role in breast cancer progression and treatment selection [41,42]. Moreover, 35 unique *ERBB2* fusions are present in ChimerDB, and at least 3 were previously described in scientific papers [43,44].

In addition, the 5′RTK fusion *FGFR2-LGSN* experimentally identified here is also absent from the above mentioned three databases. On the other hand, we found that the *LGSN* gene has been already mentioned as an *FGFR2* fusion partner in the literature [32]. We detected *FGFR2-LGSN* in an ovarian cancer sample. It has an almost fully sized *FGFR2* moiety with a preserved tyrosine kinase domain on the 5′ end. *FGFR2* is a well-studied protooncogene, and its alterations are an important biomarker for disease prognosis and treatment selection [45,46]. The *LGSN* (lengsin, lens protein with glutamine synthetase domain) gene, encoding a pseudo-glutamine synthetase protein localized to the lens, has an unknown role in the fusion functionality. *LGSN* is not normally expressed in ovarian cells, and we detected a significant increase in its exon coverage downstream to the fusion site.

The following features were found to be associated with the experimentally validated 3′ RTK fusions. First, there was a significant disbalance of RTK exon coverage by RNAseq reads downstream and upstream to the fusion site: we observed greater coverage for the downstream gene exons. This is in line with previous observations that in some clinically relevant fusion types (e.g., with *ALK* and *ABL1* genes) the 5′ partner with a highly active promoter drives the expression of the kinase domain from the 3′ moiety, which is normally transcriptionally silent in the corresponding healthy tissue [47]. Thus, our approach may be useful for identification of similar fusion events. Second, there was retention of in-frame orientation and the tyrosine kinase domain within a fusion. Third, there was a presence of the same combination of fusion partners in the specific databases of chimeric transcripts. Fourth, there was more than one identified non-redundant chimeric RNAseq read per fusion. The latter point is clearly related to the sequencing depth per library and sequencing method (we used Illumina sequencing with ribosomal depletion and random priming for 30–40 million 50 nucleotide-long single-end reads per library). Single-end reads were used because of short RNA fragments that could be extracted from FFPE. Indeed, Zhao et al. showed previously that more than 85% of RNA fragments derived from FFPE may be shorter than 100 nucleotides [48]. Biomaterials with less degraded RNA and paired-end, longer reads may result in a higher yield of directly identified chimeric sequences, but it is still important to analyze FFPE samples because this is the major way of storing cancer tissues in clinical biobanks. 

In addition to the above points, we also found that the concordance in the expression levels of the 5′-partner gene and 3′-partner exons downstream to the fusion site may be a worthwhile direction for improving the confidence of true fusion prediction. Furthermore, cancerogenesis is accompanied by numerous genetic rearrangements and “non-functional” fusions involving parts of RTK genes, but which however lack a common open reading frame for both partners and/or miss an intact tyrosine kinase domain. In certain molecular tests (e.g., FISH), such fusions could not be distinguished from the RTK chimeras with the functional tyrosine kinase, thus returning clinically misleading results which can strongly bias a personalized strategy of cancer treatment [22]. It is, therefore, important to distinguish a class of 3′RTK fusions that have in-frame tyrosine kinase domains.

The main rationale for performing this study was to find the criteria enabling reliable identification of such RTK fusions from FFPE RNAseq data without the need for experimental validation. We hope that we obtained promising results as applying a combination of at least two of the remaining three diagnostic criteria (higher number of RTK reads downstream to the fusion site, presence in the databases, and at least two reads) for in-frame fusions with intact tyrosine kinase domains leads to sensitivity of the classification of 0.86 and ~1 specificity. Automated application of the established criteria to other RNAseq datasets may enhance discovery of novel fusions. However, algorithmic prediction of exon coverage asymmetry would be the most challenging part in such analysis.

Thus, we suggest that our approach can become a basis for a new bioinformatic tool for intellectual detection of 3′RTK fusions without the need for experimental verification.

## Figures and Tables

**Table 1:** Comparative analysis of confirmed and non-confirmed fusion structural and expression features.

Sample ID	Fusion Type (5′-3′)	ORF Preserved (Yes/No) ^1^	TK Domain Retained (Yes/No) ^2^	RTK on 5′/3′ End	Presence in Fusion DBs ^3^	Greater RTK Downstream Reads (Yes/No) ^4^	Expr. of 5′/3′ Moieties ^5^	N Reads ^6^
Experimentally confirmed RTK fusions								
AL-98	*BCR-ABL1*	Yes	Yes	3′	Yes	Yes	1.02/0.47	1
FS-1	*CCDC6-RET*	Yes	Yes	3′	Yes	Yes	0.4/0.35	3
LuC-46	*SLC34A2-ROS1 variant 1*	Yes	Yes	3′	Yes	Yes	24.53/5.29	1
LuC-46	*SLC34A2-ROS1 variant 2*	Yes	Yes	3′	Yes	Yes	23.29/6.09	5
LuC-46	*SLC34A2-ROS1 variant 3*	Yes	Yes	3′	Yes	Yes	23.29/5.87	4
LuC-46	*SLC34A2-ROS1 variant 4*	No	Yes	3′	Yes	Yes	23.29/5.93	1
OC-11	*FGFR2-LGSN*	Yes	Yes	5′	No *	ND	0.72/0.43	6
OC-11	*RPS24-FGFR2*	Yes	No	3′	No	No	5.74/0.29	5
BC-105	*FBXL20-ERBB2*	No	Yes	3′	Yes	Yes	1.28/11.77	18
BC-105	*ATP2B1-ERBB2*	Yes	Yes	3′	No	No	0.41/12.41	8
Experimentally non-confirmed RTK fusions								
GC-30	*ABL1-ALDH1A2*	Yes	No	5′	No	ND	0.04/0.43	1
LuC-71	*NTRK2-AL157886.1*	No	No	5′	No	ND	NA	1
LuC-11	*KIF27-NTRK2*	Yes	Yes	3′	No	No	0.02/0.67	1
LuC-19	*NTRK2-USP47*	No	No	5′	No	ND	0.83/0.24	1
LuC-81	*ETNK1-NTRK2*	No	No	5′	No	ND	0.22/6.36	1
XC-1	*FGFR2-NFYC*	No	No	5′	No	ND	0.33/0.31	1
OC-15	*ABL1-FNIP2*	Yes	No	5′	No	ND	0.45/0.8	1
AL-44	*FGFR1-AZIN1*	No	No	5′	No	ND	0.15/0.6	1
AL-7	*RET-GART*	No	No	5′	No	ND	0.016/0.48	1
J1	*RBM18-ABL1*	No	No	3′	No	No	1.94/1.9	1
A549	*FGFR1-BRF1*	Yes	No	5′	No	ND	1.21/0.52	1
SkC-1	*ABL1-CD59*	No	No	5′	No	ND	0.05/3.58	1
BC-59	*FGFR1-REPS2*	No	No	5′	No	ND	0.55/1.5	1
EpS-1	*HMBOX1-FGFR1*	No	No	3′	No	No	0.34/5.89	1
PC-24	*DOCK1-FGFR2*	Yes	Yes	3′	No	No	0.12/0.023	1
**Overall specificity**	All confirmed vs. non-confirmed	**0.67**	**0.87**	**NA**	**1**	**1**	**NA**	**1 (1/>1)**
**Overall sensitivity**	All confirmed vs. non-confirmed	**0.8**	**0.9**	**NA**	**0.7**	**0.78**	**NA**	**0.7 (1/>1)**
Putative RTK fusions that were not experimentally investigated by RT-PCR								
TC-123	*NTRK2-MLPH*	No	No	5′	No	ND	0.97/0.22	1
N-63	*CNTNAP3-NTRK2*	No	No	3′	No	Yes	0.88/0.24	8
RAIR-4	*NCOA4-RET*	Yes	Yes	3′	Yes	Yes	0.69/0.11	1
TC-32	*NTRK2-IARS1*	Yes	No	5′	No	ND	2.55/0.74	1
TC-12	*ARHGAP12-ALK*	No	Yes	3′	No	Yes	0.46/0.02	1
BT-24	*NTRK2-ADAM32*	Yes	Yes	5′	No	ND	0.23/0.006	1
BC-47	*FGFR2-RET*	Yes	Yes	both	No	No	0.06/1.36	1
FFPE_4-2	*ZNF135-FGFR2*	Yes	Yes	3′	No	No	0.06/0.46	1
AL-84	*AGRN-FGFR3*	No	Yes	3′	No	Yes	0.82/0.17	1
FFPE-5	*AC016907.2-ALK*	No	Yes	3′	No	No	NA	1
INI-1	*KIF5C-NTRK3*	Yes	No	3′	No	Yes	1.17/0.19	1

^1^ Presence of preserved open reading frame. ^2^ Presence of preserved tyrosine kinase domain. ^3^ Presence of the chimeric transcript with the same fusion partners in ChimerDB (http://www.kobic.re.kr/chimerdb/ accessed on 21 June 2022), ChiTaRS (http://chitars.md.biu.ac.il/ accessed on 21 June 2022), and TumorFusions (https://tumorfusions.org/ accessed on 21 June 2022) databases (searched 15 June 2022). ^4^ Statistically significantly greater RTK RNAseq exon coverage downstream to the fusion site (*t*-test, *p*-value < 0.05 and/or the coverage of the exons downstream to the fusion site in sample with fusion was higher than 95th percentile of the same exons’ mean coverage in the control group, while the coverage of upstream exons was fitting within the 95% confidence interval (CI) of exon coverage level in samples without fusions), assessed only for the 3′RTK fusions; “ND” stands for the 5′RTK fusions. ^5^ Averaged expression levels of 5′- and 3′ fusion moieties calculated as RNAseq read counts normalized on lengths of exons. ^6^ Number of chimeric RNAseq reads. * FGFR2–LGSN transcript is absent from ChimerDB, ChiTaRS, and TumorFusions databases but was previously published in [32].

## Data Availability

Not applicable.

## Supplementary Materials

The following supporting information can be downloaded at: https://www.mdpi.com/article/10.3390/biomedicines10081866/s1, Table S1: Patients samples description; Table S2: The description of patients biosamples in experimental dataset; Table S3: Oligonucleotide RT-PCR primer sequences; Table S4: TRK chimeric reads found by primary STAR-Fusion analysis.
