# Supplementary material for: Experimentally Deduced Criteria for Detection of Clinically Relevant Fusion 3′ Oncogenes from FFPE Bulk RNA Sequencing Data
Source: Biomedicines. 2022 Aug 2;10(8):1866. doi: 10.3390/biomedicines10081866 (PMC9405289; doi:10.3390/biomedicines10081866)
Supplement: Supplementary file 1 [file biomedicines-10-01866-s001.zip › TableS2_The description of patients biosamples in experimental dataset.pdf]

Table S2. The description of patients biosamples in experimental dataset.

Sample ID	Putative RTK fusion type	Gender	Age	Cancer type	TNM	Grade
AL-44	*FGFR1-AZIN1*	male	2	Acute lymphoblastic leukemia	NA	NA
AL-7	*RET-GART*	female	2	Acute myeloid leukemia	NA	NA
AL-98	*BCR - ABL1*	male	5	Acute lymphoblastic leukemia	NA	NA
BC-105	*ATP2B1-ERBB2*	female	52	Breast cancer	T2N3M0	G3
	FBXL20-ERBB2					
BC-59	*FGFR1-REPS2*	female	61	Breast cancer	T1N1M0	G3
EpS-1	*HMBOX1-FGFR1*	male	60	Epithelioid sarcoma	T4bN0M0	G3
FS-1	*CCDC6 - RET*	female	58	Breast fibrosarcoma	NA	G3
GC-30	*ABL1-ALDH1A2*	female	61	Pseudomyxoma peritonei	pT4bpN3pM1	G3
LuC-11	*KIF27-NTRK2*	male	52	Lung cancer (squamous cell carcinoma)	T2N2M0	NA
LuC-19	*NTRK2-USP47*	male	65	Lung cancer (squamous cell carcinoma)	T2N0M0	G2
LuC-46	*SLC34A2-ROS1*	female	48	Lung cancer (adenocarcinoma)	NA	NA
LuC-71	*NTRK2-AL157886.1*	male	60	Lung cancer (mixed type: adenocarcinoma + squamous cell carcinoma)	T2N2M0	G2
LuC-81	*NTRK2-ETNK1*	male	54	Lung cancer (squamous cell carcinoma)	T2N0M1	NA
OC-11	*FGFR2 - LGSN*	female	38	Ovarian cancer	T3cNkhM1	G3
	RPS24-FGFR2					
OC-15	*ABL1-FNIP2*	female	48	Ovarian cancer	NA	NA
PC-24	*DOCK1-FGFR2*	female	60	Pancreatic cancer	cT4N1M1	G3
SkC-1	*ABL1-CD59*	male	34	Sarcoma Kaposi	NA	NA
XC-1	*FGFR2-NFYC*	female	58	Non-gestational choriocarcinoma	TxN1M1	NA
