# Supplementary material for: Experimentally Deduced Criteria for Detection of Clinically Relevant Fusion 3′ Oncogenes from FFPE Bulk RNA Sequencing Data
Source: Biomedicines. 2022 Aug 2;10(8):1866. doi: 10.3390/biomedicines10081866 (PMC9405289; doi:10.3390/biomedicines10081866)
Supplement: Supplementary file 1 [file biomedicines-10-01866-s001.zip › TableS3_Oligonucleotide RT-PCR primer sequences.pdf]

Table S3. Oligonucleotide RT-PCR primer sequences

Sample ID	Putative RTK fusion type	Forward primer sequence (3'-5')	Reverse primer sequence (3'-5')
A549	*FGFR1 - BRF1*	*TCGTCCTCTGTGTCCTCCTC*	*TGGTCACAGCCACACTCT*
AL_7	*RET - GART*	*GCTGAATTTTCTTGACCAGCATATTTCCA*	*GGGGCTGCGTCTGCTGTT*
AL_98	*BCR - ABL1*	*CTGAGGCTCAAAGTCAGAT*	*ACCAACTCGTGTGTGAAA*
BC_105	*FBXL20-ERBB2*	*ACTCTTTGCTCAGGGAGTAGCTTT*	*GTTCTCAAATAGTGATGAAGCTGTAATCAA*
BC_105	*ATP2B1-ERBB2*	*ATGTCCAGGTGGGTCTCGG*	*ACGAAAAATACAGGAAAGCTATGGAGATG*
BC_59	*FGFR1 - REPS2*	*GGTCTTGGAGGGAGCGCA*	*GCTGGTCACAGCCACACTC*
EpS_1	*HMBOX1-FGFR1*	*GGCTCCACATCTCCATGGATACTCCACAG*	*GGAACCCGCCGGGGAGA*
FS_1	*CCD6 - RET*	*ATTCGCCTTCTCCTAGAG*	*GCTGGAGACCTACAAACTGA*
GC_30	*ABL1 - ALDH1A2*	*TGGGATGATCTGTCCACAC*	*CAAATCCAAGAAGGGGCTG*
J_1	*RBM18 - ABL1*	*AAAGGCTTGGTGGATTTCA*	*TGATAAGATTCTTCCAATCAG*
LuC_11	*AL353743.1 - NTRK2*	*TAGACTTTCCTTCCTCCAC*	*CAGATGATGGTACAGGAAAAC*
LuC_19	*NTRK2 - USP47*	*TTCACTGTCTTTGAATTAGTAGTATCTTGTATAATACAT*	*GCGTTCGCGCACACCCT*
LuC_46	*SLC34A2 (exon 4)-ROS1(exon 33)*	*CACTGTATTGAATTTTTACTCCCTTCTAGTAAT*	*TTTTTCGTGTGCTCCCTGGAT*
LuC_46	*SLC34A2 (exon 4)-ROS1(exon 34)*	*AAATGTCATCTTCCACCTTAAATTCT*	*CTCTACTTTTTCGTGTGCTCCCT*
LuC_46	*SLC34A2 (ex 4)-ROS1(ex 35)*	*TCCAACTATAATAGTAAGTATGAAACTTGT*	*TTTCTCTACTTTTTCGTGTGCTC*

LuC_46	*SLC34A2 (exon 5)-ROS1(exon 29)*	*CCAACAAAGGGTTGGACATAATAGA*	*ACATTTACCACCAGGAAAAGAGA*
LuC_71	*NTRK2 - AL157886.1*	*GCACAGAGAGTTGGAAGGCGA*	*AATGAGTATGGGAAGGATGAGAAACAGA*
LuC_81	*NTRK2- ETNK*	*CGTTGATGATTTCTAACCTTTTCTGGTTTGC*	*GGCCCTGAGCCTCCTGC*
OC_11	*FGFR2 -LGSN*	*CTTAATGTGTTCATGCTGTTG*	*CAGTTGGTAGAAGACTTGG*
OC_11	*RPS24 - FGFR2*	*GTCAGGGTAACTAGGTGAATACTGT*	*AAGAAAGTCAGGGGGACTGCAAA*
OC_15	*ABL1 - FNIP2*	*CACTCCATTTATCCGTGTCTGCGA*	*GGCGTGTGGAAGAAATACAGCCT*
PC_24	*DOCK1-FGFR2*	*CATCCAGAAACTCTACTGCTTGATCGAAA*	*CAAAATCTTCCGCACCATCGGTGTC*
SkC_1	*ABL1 - CD59*	*AGCAGCCCGAACAGGACA*	*TCAAAGGAGCAGGGAAGAAGGAA*
XC_1	*FGFR2 - NFYC*	*GGCTTTGCTGGGCATCAC*	*ATGGCAACCTTGTCCCTG*
